# Role of Finerenone in Duchenne Muscular Dystrophy in a 45-Year-Old Man: Outcome and Brief Review

**DOI:** 10.1016/j.jaccas.2026.109526

**Published:** 2026-07-29

**Authors:** Jake Hillyer, John L. Jefferies, Marc A. Silver

**Affiliations:** a Department of Cardiovascular Disease, University of Arizona College of Medicine–Phoenix, Phoenix, Arizona, USA; b University of Memphis, Memphis, Tennessee, USA; c Banner University Medical Center Advanced Heart Failure, Heart Transplant and Mechanical Circulatory Support Program, Phoenix, Arizona, USA

**Keywords:** cardiomyopathy, Duchenne muscular dystrophy, finerenone, heart failure, mineralocorticoid receptor antagonist, myocardial fibrosis

## Abstract

**Background:**

Duchenne muscular dystrophy (DMD) is an X-linked neuromuscular disorder characterized by the absence of dystrophin, leading to progressive skeletal and cardiac muscle degeneration. Cardiomyopathy is a leading cause of morbidity and mortality in patients with DMD, often manifesting as a dilated cardiomyopathy with myocardial fibrosis and arrhythmia. Mineralocorticoid receptor antagonists, including the nonsteroidal agent finerenone, have emerged as disease-modifying therapies because of their antifibrotic, anti-inflammatory, and cardioprotective properties.

**Case Summary:**

A 45-year-old man with DMD and progressive nonischemic cardiomyopathy was initiated on finerenone in addition to guideline-directed medical therapy. Over 16 months, he demonstrated improvement in left ventricular ejection fraction and ventricular remodeling without adverse effects.

**Discussion:**

Finerenone is a nonsteroidal mineralocorticoid receptor antagonist with potent antifibrotic and anti-inflammatory properties. This case highlights its potential role in DMD cardiomyopathy, where fibrosis is central to disease progression.

**Take-Home Message:**

Finerenone may represent a novel therapy in DMD-associated cardiomyopathy and warrants further investigation for use in this population.

## Visual Summary

**Figure:**
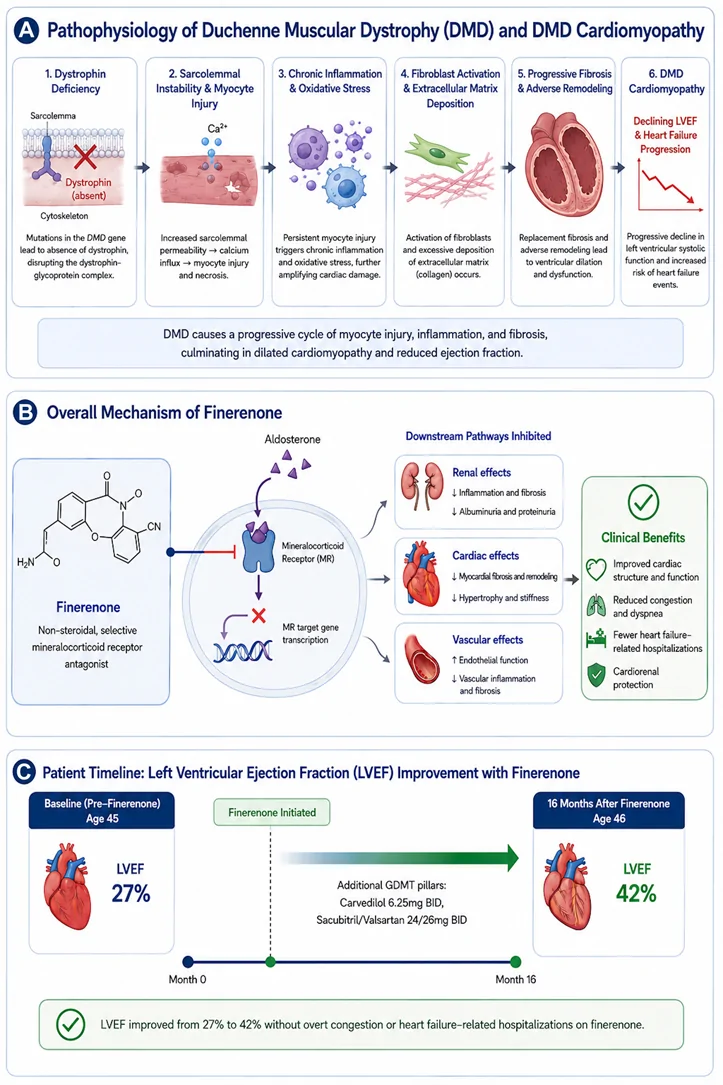

**Visual Summary:**
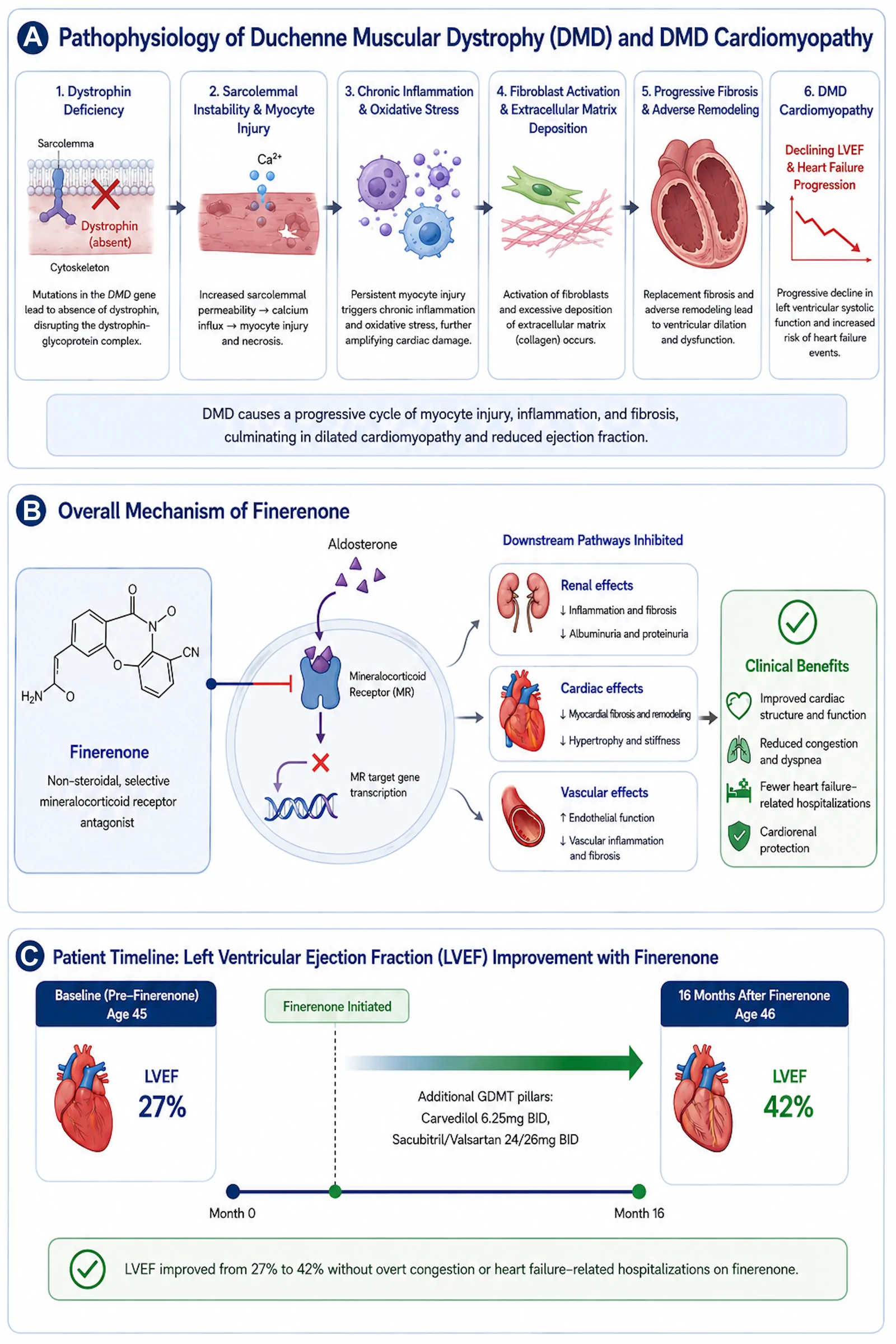
Duchenne Muscular Dystrophy Cardiomyopathy, Mechanism of Finerenone, and Clinical Course (A) Pathophysiologic cascade of Duchenne muscular dystrophy (DMD) cardiomyopathy. Loss of dystrophin results in sarcolemmal instability, increased calcium influx, chronic inflammation, oxidative stress, fibroblast activation, extracellular matrix deposition, and progressive myocardial fibrosis, culminating in adverse ventricular remodeling and systolic dysfunction. (B) Mechanism of action of finerenone: nonsteroidal selective mineralocorticoid receptor antagonist that inhibits mineralocorticoid receptor–mediated transcription of proinflammatory and profibrotic pathways. Downstream effects include attenuation of cardiac fibrosis and remodeling, reduction in vascular inflammation, and cardiorenal protection. (C) Clinical timeline demonstrating improvement in left ventricular ejection fraction (LVEF) following initiation of finerenone in a 45-year-old man with DMD-associated cardiomyopathy. BID = twice a day; GDMT = guideline-directed medical therapy.

## Case Presentation

### History of Present Illness

A 45-year-old man with Duchenne muscular dystrophy (DMD) was referred to the advanced heart failure clinic for longitudinal management of progressive cardiomyopathy. Before his presentation, serial transthoracic echocardiograms had demonstrated progressive deterioration of left ventricular systolic function with left ventricular ejection fraction declining from approximately 40% at age 42 to 27% by age 45. Of note, despite his progressive structural heart disease, he had experienced no heart failure–related hospitalizations and remained clinically stable without overt congestive symptoms (eg, worsening hypoxemia, progressive dyspnea, or peripheral edema) on guideline-directed medical therapy.

## Past Medical History

The patient's medical history included DMD diagnosed at age 3 by skeletal muscle biopsy, permanent atrial fibrillation status-post atrioventricular node ablation and permanent pacemaker placement at age 30, dilated cardiomyopathy complicated by ventricular tachycardia status-post device upgrade to cardiac resynchronization therapy with implantable cardioverter-defibrillator (CRT-D) implantation at age 37, obstructive sleep apnea, and chronic hypoxemic respiratory failure requiring tracheostomy at age 37 with chronic ventilator dependence. The patient's guideline-directed medical therapy consisted of carvedilol 6.25 mg twice daily and sacubitril/valsartan 24/26 mg twice daily in addition to furosemide 20 mg daily. The remainder of his home medications were theophylline 200 mg scheduled once daily, nebulized arformoterol 15 μg/2 mL scheduled twice daily, and nebulized albuterol-ipratropium 3 mL inhaled every 6 hours as needed for shortness of breath.

## Differential Diagnosis

The differential diagnosis for his worsening left ventricular systolic function included progressive DMD-associated cardiomyopathy, tachycardia-mediated cardiomyopathy, and device-related dyssynchrony.

## Investigations

Vital signs on presentation were a blood pressure of 92/68 mm Hg with a heart rate of 90 beats/min, and a respiratory rate of 18 breaths per minute with an oxygen saturation of 98%. Laboratory evaluation demonstrated depressed but stable serum creatinine (<0.6 mg/dL; normal range 0.68-1.37 mg/dL), normokalemia (4.1 mmol/L; normal range 3.6-5.3 mmol/L), and elevated N-terminal pro–B-type natriuretic peptide (224 pg/mL; normal <124 pg/mL). Iron panel and thyroid studies were also normal.

**Figure 1:**
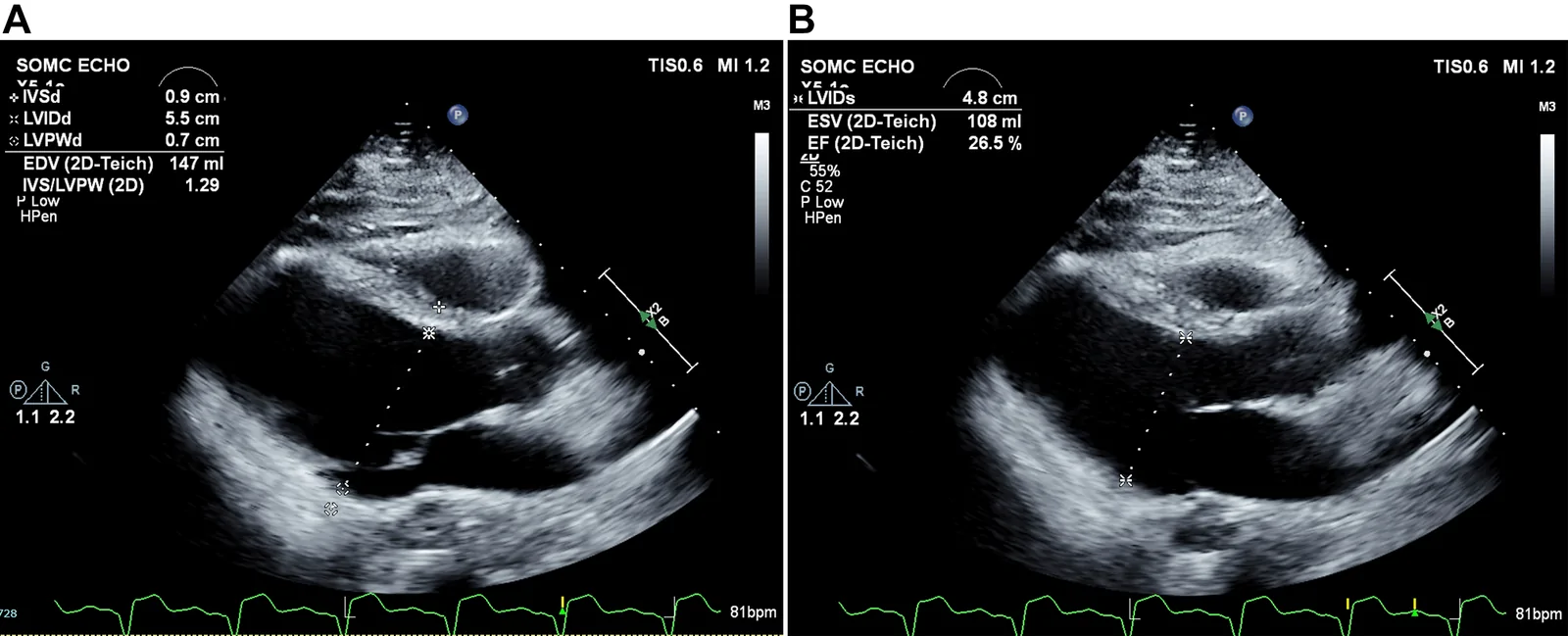
Baseline Transthoracic Echocardiogram in the Parasternal Long-Axis View Demonstrating Left Ventricular Ejection Fraction of 27% Before Initiation of Finerenone (A) End-diastole with left ventricular internal dimension at end-diastole (LVIDd) of 5.5 cm and (B) end-systole with left ventricular internal dimension at end-systole (LVIDs) of 4.8 cm. EDV = end-diastolic volume; EF = ejection fraction; ESV = end-systolic volume; IVSd = interventricular septum thickness at end-diastole; LVPWd = left ventricular posterior wall thickness at end-diastole.

Transthoracic echocardiogram (Figure 1) revealed a left ventricular ejection fraction of 27% by biplane method of discs with global hypokinesis and akinesis of the inferolateral wall, a normal left ventricular internal dimension at end-diastole of 5.5 cm (normal range 4.2-5.8 cm) and severely abnormal left ventricular internal dimension at end-systole of 4.8 cm (normal range 2.5-4.0 cm).

CRT-D device interrogation showed normal device function programmed to dual-chamber pacing, dual-chamber sensing, and dual-chamber response with 99% biventricular pacing. There was one episode of sustained ventricular tachycardia successfully treated with antitachycardia pacing therapy within 30 days before presentation to the advanced heart failure clinic.

## Management

As part of ongoing optimization, spironolactone was initiated as an adjunctive mineralocorticoid receptor antagonist (MRA) strategy. At clinic follow-up 1 month later, the patient reported intermittent symptomatic hypotension and was noted to have a systemic blood pressure of 78/59 mm Hg with repeat measurements in the 70s/50s. Because of a concern for intolerance to spironolactone and a perceived clinical benefit of using a nonsteroidal aldosterone antagonist, finerenone 10 mg daily was added to his regimen. No changes were made to his CRT-D device programming.

## Outcome and Follow-Up

**Figure 2:**
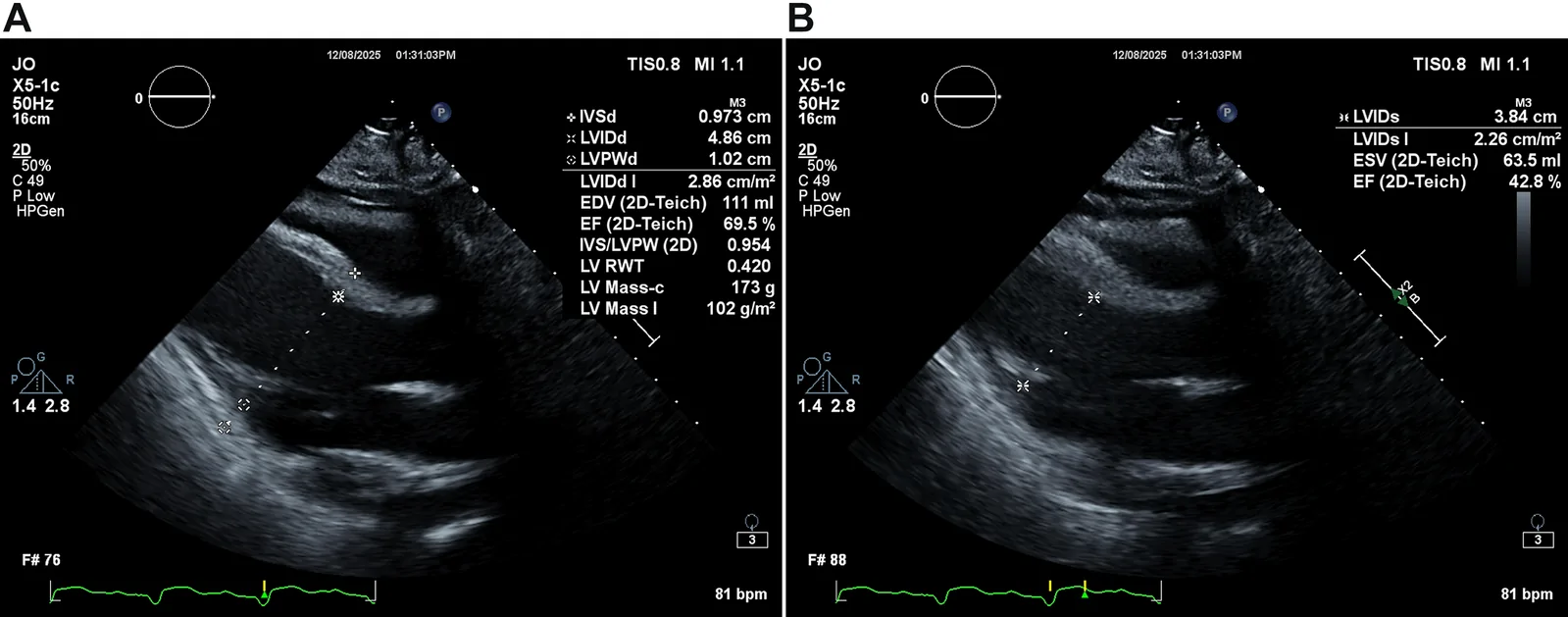
Parasternal Long-Axis View of the Transthoracic Echocardiogram Demonstrating Improvement of Left Ventricular Ejection Fraction of 42% After Initiation of Finerenone (A) End-diastole with left ventricular internal dimension at end-diastole (LVIDd) of 4.9 cm and (B) End-systole with left ventricular internal dimension at end-systole (LVIDs) of 3.8 cm. EDV = end-diastolic volume; ESV = end-systolic volume; IVSd = interventricular septum thickness at end-diastole; LVPWd = left ventricular posterior wall thickness at end-diastole; LV = left ventricular; RWT = relative wall thickness.

The patient tolerated finerenone well without hyperkalemia, changes to renal function, or symptomatic hypotension. After 1 year of therapy, there had been no changes to his functional status, and he remained free of heart failure–related symptoms or hospitalizations. During this time, no additional changes were made to his guideline-directed medical therapy as he remained on stable doses of carvedilol 6.25 mg twice daily and sacubitril/valsartan 24/26 mg twice daily in addition to furosemide 20 mg daily. Repeat transthoracic echocardiogram (Figure 2) 16 months after initiation of finerenone demonstrated an improvement in left ventricular ejection fraction to 42% by biplane method of discs. Although maintained within the normal range, left ventricular internal dimension at end-diastole improved from 5.5 cm to 4.9 cm. The severely elevated left ventricular internal dimension at end-systole of 4.8 cm improved into the normal range at 3.8 cm. He is currently awaiting cardiac magnetic resonance imaging for further evaluation of myocardial remodeling and fibrosis.

## Discussion

DMD affects approximately 1 in 5,000 live male births and is caused by mutations in the dystrophin gene.^1^ Dystrophin is a structural protein that acts as a molecular shock absorber, linking the intracellular actin cytoskeleton to the extracellular matrix via the dystrophin-associated glycoprotein complex; this complex protects muscle fibers from damage during contraction and relaxation. In DMD, the absence of functional dystrophin leads to sarcolemmal instability, rendering myocytes susceptible to contraction-induced injury.

In the cardiac muscle, repeated mechanical stress results in increased membrane permeability and calcium influx, activation of proteolytic enzymes and mitochondrial dysfunction, myocyte necrosis and apoptosis, and replacement of myocardium with fibrofatty tissue.^2^^,^^3^ These processes preferentially affect the subepicardial regions of the inferolateral left ventricular wall, a hallmark imaging feature in DMD cardiomyopathy. Over time, progressive myocardial fibrosis leads to ventricular dilation, systolic dysfunction, impaired diastolic relaxation, and an increased risk of ventricular arrhythmias.

DMD disease progression is further exacerbated by chronic activation of the renin-angiotensin-aldosterone system, which promotes sodium retention, oxidative stress, inflammation, and fibroblast activation and accelerates adverse cardiac remodeling in DMD. Although mineralocorticoid receptor antagonism is well established as adjunctive therapy in heart failure with reduced ejection fraction, its role in DMD may be particularly compelling given the central contributions of myocardial fibrosis and inflammation to disease progression.^4^, ^5^, ^6^, ^7^ These pathophysiologic features provide a rationale for targeting aldosterone signaling as a means of modifying disease trajectory.

MRAs may benefit patients with DMD through several complementary mechanisms, including reduction of myocardial fibrosis via inhibition of aldosterone-mediated fibroblast activation, attenuation of inflammation through suppression of proinflammatory cytokines and macrophage infiltration, and improvement in ventricular remodeling by limiting fibrosis and oxidative stress.^6^^,^^7^ By reducing fibrotic substrate and electrical remodeling, MRAs also may decrease ventricular arrhythmia risk. When combined with angiotensin-converting enzyme inhibitors or angiotensin receptor blockers, MRAs provide more complete neurohormonal blockade in a disease characterized by chronic renin-angiotensin-aldosterone system activation.^6^

Finerenone is a nonsteroidal, highly selective MRA with distinct pharmacodynamic properties. Unlike steroidal MRAs (ie, spironolactone, eplerenone), finerenone has a balanced tissue distribution between the heart and kidneys and induces a different conformational change in the mineralocorticoid receptor, leading to potent anti-inflammatory and antifibrotic effects with less renal sodium channel activation.^8^ Finerenone has been shown to reduce cardiac fibrosis, oxidative stress, and inflammatory gene expression in experimental models.^9^ Clinically, it is associated with a lower risk of hypotension, antiandrogenic side effects, and hyperkalemia compared with traditional MRAs at equivalent antifibrotic potency,^10^ making it an attractive potential therapy for chronic cardiomyopathies such as DMD.

Collectively, these effects support the early use of MRAs—especially more selective agents such as finerenone—in the management of DMD cardiomyopathy, even before overt heart failure develops. The present case illustrates the complexity of advanced cardiomyopathy in DMD and highlights the potential role of nonsteroidal mineralocorticoid receptor antagonism in long-term disease stabilization. To our knowledge, this is the first documented use of finerenone in a patient with DMD, opening the door to future investigation into its safety, efficacy, and ultimate role in this population. Further studies are needed to define the optimal timing, dosing, and selection of MRAs in patients with DMD.

## Conclusions

DMD-associated cardiomyopathy is driven by dystrophin deficiency, myocardial injury, and progressive fibrosis. MRAs, including finerenone, target key neurohormonal and fibrotic pathways involved in disease progression. Their physiologic effects provide a strong rationale for use as part of a comprehensive, early cardioprotective strategy in patients with DMD.

## Funding Support and Author Disclosures

The authors have reported that they have no relationships relevant to the contents of this paper to disclose.

**Take-Home Messages**

• Myocardial fibrosis is a major driver of Duchenne muscular dystrophy cardiomyopathy, making early neurohormonal blockade—including mineralocorticoid receptor antagonism—a rational therapeutic strategy.
• This case provides the first clinical evidence supporting the feasibility of finerenone therapy in Duchenne muscular dystrophy cardiomyopathy and highlights the need for prospective evaluation of nonsteroidal mineralocorticoid receptor antagonists in this population.
